# Type III Monteggia Injury With Ipsilateral Distal Forearm Fracture in a Child: A Case Report

**DOI:** 10.3389/fped.2021.805985

**Published:** 2022-01-31

**Authors:** Chao Gao, Jing Hua Sun, Hua Jiang Zheng, Yong Yao Wu, Jin Cao

**Affiliations:** ^1^Department of Orthopedics, Ningbo Sixth Hospital, Ningbo, China; ^2^Department of Pathology, Ningbo Diagnostic Pathology Center, Ningbo, China; ^3^Nephrology Department, Ningbo Medical Center Lihuili Eastern Hospital, Ningbo, China

**Keywords:** Monteggia injury, ipsilateral distal forearm fracture, bipolar fracture, child, operative treatment

## Abstract

Monteggia fracture refers to breakage of the upper third of the ulna combined with dislocation of the radial head. It often occurs in children and adolescents and represents a combined injury. Fracture of the distal forearm is among the most common trauma suffered by children. However, distal forearm fractures have rarely been reported as having an association with Monteggia fractures. We report on a 9-year-old boy diagnosed with a type III Monteggia fracture combined with a distal forearm fracture. He underwent surgery and received rehabilitation training 1 month later. He was followed-up for 1 year. The affected limb functioned well with no sign of radial head dislocation.

## Introduction

A Monteggia fracture is one in which the upper third of the ulna breaks while simultaneously a dislocation of the radial head occurs, representing a combined injury. It is uncommon in children, accounting for only 0.4% of the fractures in childrens' forearms (1). It was first reported by Monteggia, an Italian surgeon in 1814. In 1967, Bado termed this type of injury a Monteggia fracture, with 4 classifications that depend on the direction of the dislocation of the radial head (2). Of these injuries, type I (59%) and type III (26%) are the most common. Because of the high rate of misdiagnosis, the complex mechanism of injury and presentation of challenging complications, Monteggia fracture has been the focus of attention of researchers. Although such fractures have become increasingly recognized in the orthopedics community, the fracture itself remains a challenging clinical phenomenon. In pediatric patients, fractures surrounding the elbow and wrist joints are common. Distal forearm fractures are one of the most common injuries in children, and its incidence is relatively high, accounting for approximately 32.9% of the fractures in children, with a peak incidence at 9.9 years of age (3). However, ipsilateral elbow and wrist fractures are rare (4). The present article reports the case of a 9-year-old boy who was diagnosed with a Monteggia fracture (Bado type III) combined with a fracture of the ipsilateral forearm.

## Case Report

**Figure 1:**
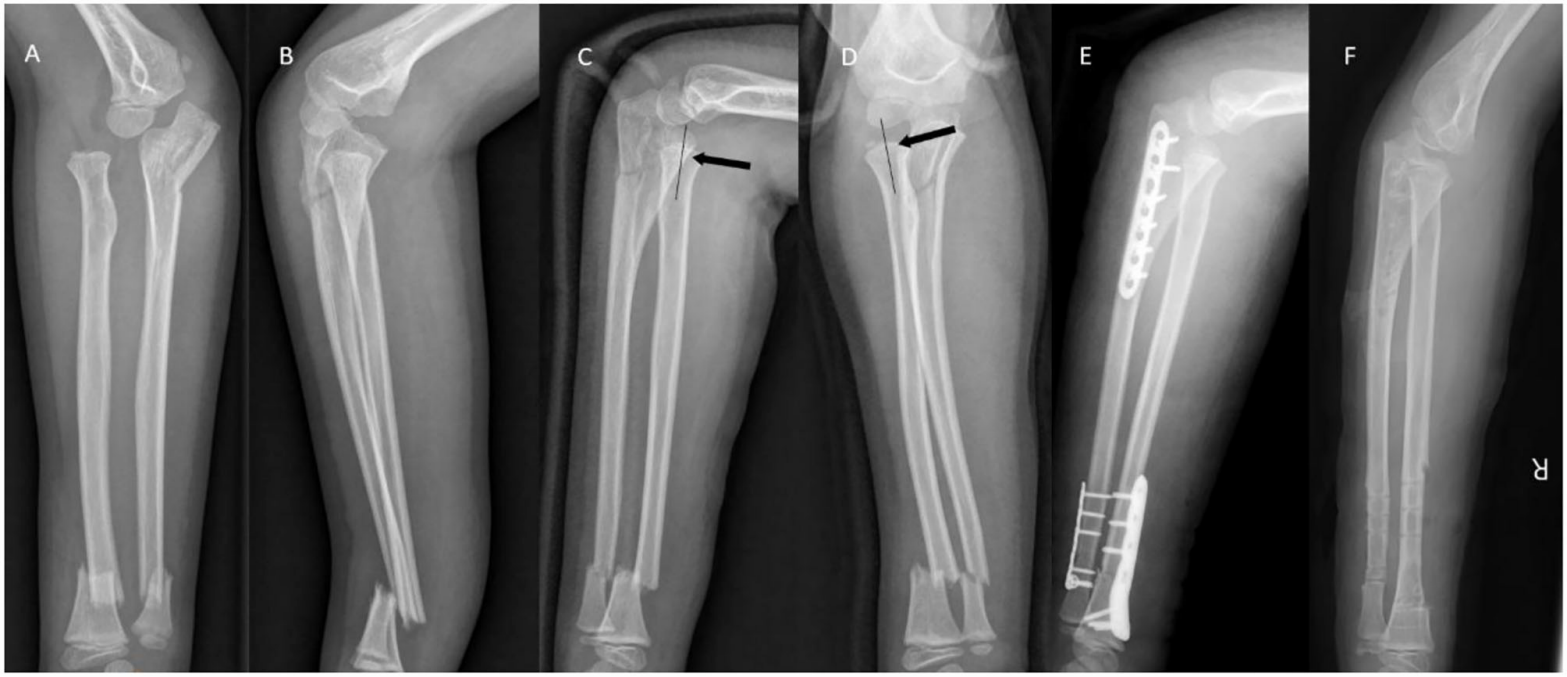
**(A,B)** X-ray images at initial examination. Bado type III Monteggia fracture and ipsilateral distal forearm fracture were observed. **(C,D)** Following initial traction, the radial axis did not pass the midpoint of the humeral capitulum (black arrow). **(E)** Intraoperative radiographs demonstrated good alignment of the fracture. **(F)** One year after surgery.

A 9-year-old boy complained of pain and swelling with restricted mobility in his right forearm. Three hours earlier, he had accidentally fallen 2 meters from a platform while playing. A pulse from the radioulnar artery was palpable but the right wrist and elbow joints were clearly distorted and swollen, with painful and restricted movement. The child was also unable to perform dorsiflexion of the right first to third fingers. X-ray films indicated fractures of the distal ulna and radius and proximal ulna, with lateral dislocation of the radial head (Figures 1A,B). Considering that the patient displayed symptoms of nerve compression, manual reduction was performed as quickly as possible, with the right elbow joint and forearm placed in a cast. Numbness in the right hand improved significantly after reduction, but dorsiflexion function was poor. After reviewing additional X-rays, it was found that radial head dislocation remained, with poor alignment of the right forearm fracture reduction (Figures 1C,D). Four days later, the patient underwent open reduction of the fracture with internal fixation. Surgery was performed following brachial plexus anesthesia, in which the patient was placed in a supine position while a pneumatic tourniquet was utilized. Briefly, two longitudinal skin incisions (~3cm in length) were created aseptically, with the fracture of the distal ulna and the radius at the center. After separating the skin and fascia, layer by layer, the ends of the fracture were exposed. The incarcerated soft tissue was reduced and the fractured end fixed with miniplates and screws. Intraoperative fluoroscopy demonstrated that the fractured end had been reduced correctly.

A posterior median incision of the elbow joint was created to reduce the proximal ulnar fracture. A compressive comminuted fracture of the proximal ulna was observed. Following removal of the bone fragments, the fracture was fixed with a compression plate. The forearm was subsequently supinated and the elbow joint flexed to reduce the radial head. Intraoperative fluoroscopy indicated that the humeroradial joint was well-positioned, and dislocation of the radial head was corrected (Figure 1E). Following surgery, the forearm was immobilized with an above-elbow splint and the patient was discharged from hospital a week later. After discharge, the patient was reexamined in the outpatient department. The plaster cast was removed in the outpatient treatment room after 4 weeks. The forearm could be pronated by 60°, or supinated by 50°, and ~60° of flexion or extension of the right elbow joint could be achieved (range: 60–120°). The Broberg-Morrey score was 54 points. Dorsiflexion of the fingers had gradually recovered, and was fully restored after 2 months. Following two months of functional exercise, the patient's forearm range of motion had returned to 90° pronation, 80° supination, and 110° movement of the right elbow (range: 0–110°) with a Broberg-Morrey score of 88 points. The plate was removed 1 year after surgery (Figure 1F).

## Review of the Current Literature

Monteggia fracture combined with ipsilateral distal forearm fracture is a rare injury, for which the literature is limited. As described by Odena (5), this type of injury is also known as a bipolar fracture of the forearm. A search of the literature identified 9 previously published cases that were similar (Table 1). Patient ages ranged from 5 to 12 years, with a mean of 9 years. The male to female ratio was 3.5:1. All patients sustained injuries by falling from heights ranging from 1.5 to 4.5 meters, with a mean of 2.49 meters. An interesting phenomenon was the presence of a distal forearm fracture with dorsal angulation in each patient, indicating that the wrist was in dorsiflexion and forearm in pronation at the time of injury. Injuries due to falls from a height often result in multiple fractures of the forearm that are clearly misaligned, requiring surgical treatment. Of the cases in the literature, 3 patients received conservative treatment, the remaining 6 undergoing surgery. The duration of fixation ranged from 2 to 8 weeks, with 89% (8/9) of patients having a duration ≥4 weeks, and 11% (1/9) with a duration ≤3 weeks.

**Table 1:** Overview of previous bipolar fractures of the forearm.

**Article**	**Specification of Injury**	**Case Description**	**Distal fracture fragment**	**Treatment**	**Results**
Kamudin NAF (6)	Type III Monteggia injury with ipsilateral distal end radius fracture and metaphyseal fracture of the distal ulna	A 12 year old girl fell from a tree of about 15 feet height	Dorsal dislocation	Cast for 4 weeks. The radial head was relocated using closed manipulative reduction. The distal end of the left radius and proximal ulna were fixed with K-wires	After 2 months, full flexion and extension of the elbow and wrist, with full pronation of the forearm, but limited forearm supination (0–60°).
Gaurav Mundada (7)	Type I Monteggia injury with Ipsilateral fracture of the distal radius and epiphyseal injury	A 11 year old boy fell from a tree from about 5 feet	Dorsal dislocation	Cast for 4 weeks. The distal end of the left radius was fixed with Kirschner wire. The proximal ulna was fixed with a 2.5mm plate	6 months post-operatively, elbow (0°-110°), with 30° wrist dorsiflexion and 40°plantarflexion.
Huw LM Williams (8)	Type III Monteggia injury with ipsilateral type II Salter Harris injury	A 5 year old boy fell from a tree from about 5 feet	Dorsal dislocation	Cast for 5 weeks. The radial head was relocated by closed reduction, K-wires were used to stabilize the distal radius fracture. Ulna fracture was treated non-operatively	After 6 months, full range of movement at the elbow and wrist.
Noel Peter (9)	Type I Monteggia lesion with distal radial and ulna metaphyseal fracture	A 5 year old boy fell from a height of ~2–3 m	Dorsal dislocation	Cast for 4 weeks. Manual reduction	At 12 weeks post injury, no limitation of motion in the affected joints
Asheesh Sood (10)	Type I Monteggia fracture with ipsilateral fracture of the distal radius and ulna	A 11 year old girl fell from a tree from about 1.8 m	Dorsal dislocation	Cast for 6 weeks. The radial neck was reduced with direct observation. The ulna was reduced and fixed with a six-hole dynamic compression plate. The distal wrist fracture was stabilized with K-wires	After 7 months, complete range of motion in both elbow and wrist had been restored
A. Biyani (11)	Ipsilateral fracture of both the radius and ulna at proximal and distal metaphyseal levels	A 10 year old boy fell from a ladder	Dorsal dislocation	Cast for 5 weeks. Manual reduction	After 1 year, complete range of motion in both elbow and wrist had been restored
Hiroshi Maeda (12)	Type III Monteggia fracture with Galeazzi fracture	A 10 year old boy fell from a basketball net from about 3 m	Dorsal dislocation	Cast for 8 weeks. Manual reduction	After 3 years, no limitation of motion in the affected joints
Dhananjay Singh (13)	Type I Monteggia fracture with ipsilateral fracture of the distal forearm	A 11 year old boy fell from a window	Dorsal dislocation	Cast for 6 weeks. Ulna was fixed using a intramedullary nail. Radius fracture was fixed using K-wires	At final follow-up at 6 months, no limitation of motion in the affected joints
Takeshi Inoue (14)	Type III Monteggia Injury with ipsilateral fracture of the distal radius and ulna	A 6 year old boy fell from a climbing pole from about 3 m	Dorsal dislocation	Cast for 2 weeks. Both ulna and radius fracture fixed using K-wires	At final follow-up after 21 years, no limitation of motion in the affected joints

## Injury Mechanism

**Figure 2:**
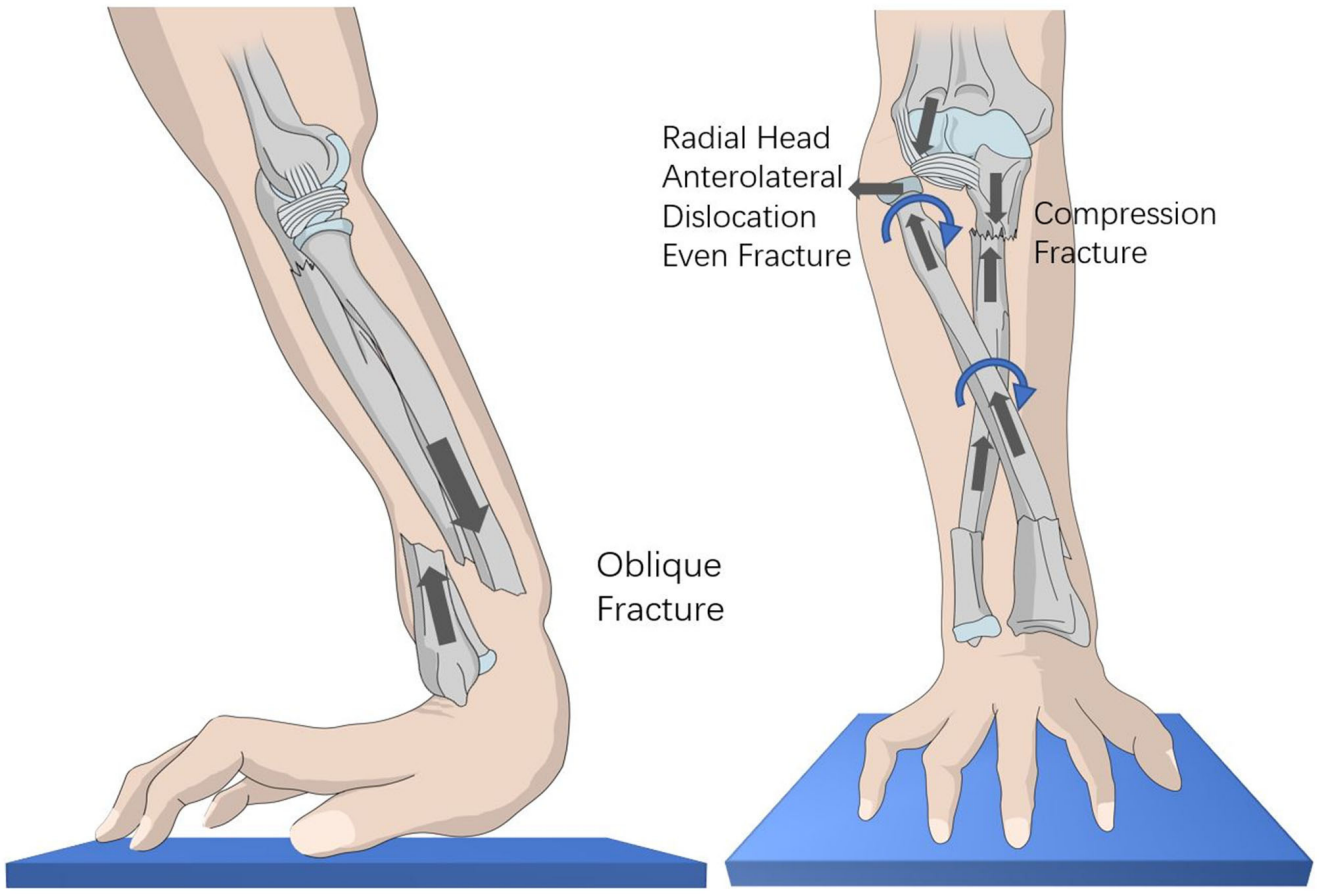
Mechanism of this type of injury.

It is often difficult to determine the exact mechanism of an injury in young children because they are often unable to communicate effectively after sustaining an injury (15). However, the type of forearm fracture observed on the X-ray images and the direction of radial head dislocation and ulnar fracture all provide indirect clues to the mechanism of injury (16). Based on our analysis of previous cases, we found that falls are a prerequisite for this type of injury. The mechanisms are as follows: 1. Strong vertical force. 2. The forearm is always pronated when a child falls to the ground with an outstretched hand (17). In this scenario, the ulna is straight and more prone to compression fracture, while the radius is inclined, force more likely to cause anterolateral dislocation of the radial head. In the present case, analysis of X-ray images revealed that vertical impact from falling had fractured the distal forearm (Figure 2A), with the force conducting upward along the radius and ulna, respectively, causing dislocation of the radial head and compression fracture of the proximal ulna. Because the radius was pronated, vertical force often results in anterolateral dislocation of the radial head, or even fracture (Figure 2B). This explains why the majority of elbow fractures are type I or III Monteggia injuries.

## Radial Nerve Injury

Radial nerve injury is the most common complication of Monteggia fractures (18). They often occur in type I and III injuries, of which type III is more common. The reason is that the radial nerve is close to the Frohse arch at the proximal end of the radial head. The arch is thinner in children, possibly causing children's nerves to be damaged more easily. Patients usually present with nerve palsy, although function is quickly restored after reduction of the radial head dislocation. In the present study, the child exhibited injury of the radial nerve, with numbness and limited dorsiflexion of fingers 1–3. After discovering that the radial head was dislocated, it was manually reduced in the emergency department, causing the numbness to disappear, but the child was still unable to perform dorsiflexion of his fingers. Finger movement gradually returned to normal over time. For most patients, function is restored within 6 to 12 weeks of an injury. Where no apparent improvement in function is observed 4 weeks after injury, electromyography can be performed to check whether the radial nerve is damaged. Such patients often require further surgical exploration.

We emphasize that emergency manual reduction of a radial head dislocation is important so that traction of the radial nerve caused by the dislocation does not result in irreversible loss of nerve function caused by long-term compression.

## Therapeutic Method

In terms of treatment, successful results have been reported with non-surgical approaches (9, 11, 12). Previous studies have demonstrated that conservative treatment is often effective in patients with stable fractures and dislocations. However, for most patients, due to the greater force causing the injury, the fractured ends are often significantly dislocated, and so surgery is required. Stable reduction of ulnar fractures and restoration of the ulnar bow is the key outcome of surgery. When the ulna is reset, the radial head can still be dislocated. This is often due to compression of the annular ligament or bone fragments, and the radial head needs to be reset while observing directly.

## Conclusion

The present article reports a case of multiple forearm fractures with radial nerve injury. After surgery, the patient recovered well. It can be concluded that satisfactory outcomes for Monteggia fracture and dislocation require early manual reduction, stable anatomical reduction of ulnar fractures, and reduction of the radial head. Although closed reduction can be achieved in the majority of such injuries in children, failure of closed reduction, as in this case, surgical fixation should be performed without hesitation.

## Data Availability Statement

The original contributions presented in the study are included in the article/supplementary material, further inquiries can be directed to the corresponding author/s.

## Ethics Statement

The parents of patient in our study were informed about the management of this special fracture. They chose operative treatment. They signed a consent form for the participation of their child in the publication and about long-term follow-up. Written informed consent was obtained from the minor(s)' legal guardian/next of kin for the publication of any potentially identifiable images or data included in this article.

## Author Contributions

HJZ, JHS, and YYW collected the data. CG wrote the first draft of the manuscript. JC contributed to interpretation of data modified this paper and approved the final version. All authors were involved in the conception of the paper.

## Conflict of Interest

The authors declare that the research was conducted in the absence of any commercial or financial relationships that could be construed as a potential conflict of interest.

## Publisher's Note

All claims expressed in this article are solely those of the authors and do not necessarily represent those of their affiliated organizations, or those of the publisher, the editors and the reviewers. Any product that may be evaluated in this article, or claim that may be made by its manufacturer, is not guaranteed or endorsed by the publisher.
